# Social Determinants of Pharmacy Deserts in Los Angeles County

**DOI:** 10.1007/s40615-020-00904-6

**Published:** 2020-10-27

**Authors:** Cheryl Wisseh, Kristin Hildreth, Jazalene Marshall, Ashton Tanner, Mohsen Bazargan, Paul Robinson

**Affiliations:** 1 Department of Clinical Pharmacy Practice, School of Pharmacy and Pharmaceutical Sciences, University of California at Irvine, Irvine, CA, USA; 2 Department of Family Medicine, College of Medicine, Charles R. Drew University of Medicine and Science, Los Angeles, CA, USA; 3 Enhanced Post Baccalaureate Certificate Program in Pre-Medicine, College of Medicine, Charles R. Drew University of Medicine and Science, Los Angeles, CA, USA; 4 Department of Biomedical Science, College of Science and Health, Charles R. Drew University of Medicine and Science, Los Angeles, CA, USA; 5 Department of Family Medicine, University of California, Los Angeles, Los Angeles, CA, USA; 6 Department of Surgery, College of Medicine, Charles R. Drew University of Medicine and Science, Los Angeles, CA, USA; 7 Department of Ophthalmology, University of California, Los Angeles, Los Angeles, CA, USA

**Keywords:** Pharmacy, Medication access, Social determinants of health, Pharmacy deserts, Population health, Public health

## Abstract

As medications are commonly used to prevent and mitigate chronic diseases and their associated complications and outcomes, limited geographic access to medications in communities that are already plagued with health inequity is a growing concern. This is especially important because low-income urban minority communities often have high prevalence and incidence of cardio-metabolic and respiratory chronic conditions. Community pharmacy deserts have been established in Chicago, New York, and other locales. In part because the definition was originally adapted from the concept of food deserts, existing studies have either utilized the distance of 1 mile or greater to the nearest community pharmacy solely, or used distance along with the same predefined social indicator thresholds that define food deserts (i.e., income and vehicle ownership), to define and identify areas as pharmacy deserts. No full analysis has been conducted of the social determinants that define and characterize medication shortage areas within a given locale, even though medication and food are usually accessed independently. Therefore, to address this gap in the literature, this study was designed to identify all potential “pharmacy deserts” in Los Angeles County based on distance alone and then characterize them by their social determinants of health (SDOH) indicators. Geographic pharmacy deserts were identified as census tracts where the nearest community pharmacy was 1 mile or more away from a tract centroid. K-means clustering was applied to group pharmacy deserts based on their composition of social determinants of health indicators. Twenty-five percent (571/2323) of LA County census tracts were pharmacy deserts and 75% (1752/2323) were pharmacy non-deserts. Within the desert areas, two statistically distinct groups of pharmacy deserts (type one and type two) emerged from the analysis. In comparison to type two pharmacy deserts, type one pharmacy deserts were characterized by a denser population, had more renters, more residents that speak English as a second language, less vehicle ownership, more residents living under the federal poverty level, more Black and Hispanic residents, more areas with higher crime against property and people, and less health professionals to serve the area. Residing in type one desert areas, potentially compounds the geographic shortage of pharmacies and pharmacy services. As such, residents in Los Angeles County pharmacy deserts might benefit greatly from equitable, innovative, community-based interventions that increase access to medications, pharmacy services, and pharmacists.

## Introduction

According to the Centers for Medicare and Medicaid Services (CMS), the USA spent $333.4 billion on prescription drugs in 2017 and this accounted for 10% of overall health care expenditures in the USA [1]. Moreover, 90% of such expenditures were for individuals that have chronic conditions. As such, there is a critical need for accessible pharmacies and pharmacy services. However, a retrospective analysis demonstrated that although there had been reasonable growth in the number of pharmacies across the USA from 2007 to 2015, a majority of pharmacies did not offer services that facilitated community prescription medication access [2]. Poor access to medications is often exacerbated in urban, rural, and racially segregated areas and such areas have been identified as pharmacy deserts nationally and internationally. [3–9]. Pharmacy closure is also a factor that contributes to the formation and existence of pharmacy deserts. Moreover, the risk of pharmacy closure is associated with reduced medication adherence and is greater for pharmacies that serve disproportionately low-income and uninsured populations [10, 11]. Pharmacy deserts might also contribute to racial/ethnic and socioeconomic disparities in medication use, which in turn may worsen racial/ethnic and socioeconomic disparities in chronic disease outcomes. In a study that examined the association between pharmacy accessibility, utilization, and cost-related underuse of prescription medications in predominantly Black and Hispanic communities of low socioeconomic status in Chicago, residents with low access to pharmacies were more likely to report cost-related underuse of prescription medications in comparison to residents with pharmacies that were located less than 1 mile from their home [12]. Cost-related non-adherence to medications is an associated consequence of the lack of economic stability, which is one of the five core areas of the social determinants of health. The other four core areas of the social determinants of health (SDOH) framework include education, health and health care, social and community context, and the neighborhood and built environment [13].

Defined as conditions in the environments in which individuals are born, live, learn, work, play, worship, and age [14], SDOH have been measured and represented by a variety of different indicators in previous works that have identified pharmacy deserts in the USA. Such variables include but are not limited to percentage of residents living below the federal poverty level (FPL), households without vehicles, percentage of residents without health insurance, home ownership, median household income, index of medical underservice (IMU) or health professional shortage area (HPSA) designation status, and crime risk scores [3, 5, 7, 15]. Furthermore, it can be inferred from these works that the forces of structural inequity that create disparities in the social determinants of health also contribute to the formation of some pharmacy desert areas [16]. The current literature contains two broad methodological approaches for identifying and characterizing pharmacy deserts. The first approach utilizes the distance of 1 mile or more to a community pharmacy to define and identify communities as pharmacy deserts and then reports on the SDOH indicators found in these areas [15]. This approach leads to conflation between urban, rural, and suburban deserts. The second approach utilizes predefined SDOH indicators (i.e., poverty and vehicle ownership) along with the distance of 1 mile or more to a community pharmacy to pre-identify areas as pharmacy deserts. For example, in Chicago, the pre-identified pharmacy desert criteria were (1) greater than 1 mile through the street network to a community pharmacy, (2) greater than 20% of residents living below FPL, and (3) racial and ethnic composition and segregation of the community [3]. This approach uses the same defining social characteristics as “food deserts” and potentially excludes areas that do not meet these predefined “thresholds” but whose residents might actually lack access to pharmacy services.

Recently, Kolak and colleagues (2020) developed four principal SDOH indices: the socioeconomic advantage index, the limited mobility index, the urban core opportunity index, and the mixed immigrant cohesion and accessibility index [14]. Each principal component accounted for 40%, 13.4%, 9.6%, and 8.1%, respectively, and 71% overall of the variance in 15 SDOH analysis variables across all census tracts in the USA, thus demonstrating heterogeneity in SDOH across most geographical areas in the USA. It is crucial to note that areas of profound socioeconomic disadvantage were found in small and dense census tracts in Los Angeles, California; a locale whose racial and socioeconomic disparities in chronic disease outcomes are well described in the literature [16, 17]. As medications are commonly used to prevent and mitigate chronic diseases and their associated complications and outcomes, limited geographic access to medications in communities that are already plagued with health inequity is a growing concern. This is especially important because low-income minority communities in Los Angeles County often have high prevalence and incidence of cardio-metabolic and respiratory chronic conditions. Racial and ethnic groups that were once minorities collectively now make up the majority in Los Angeles County. Moreover, the racial and ethnic composition of the county is 48.7% White, 11.0% African American, 0.8% Native American, 10.0% Asian, 0.3% Pacific Islander, 23.5% from other races, and 4.9% from two or more races. Almost 44.6% of the population are Hispanic or Latino of any race [18]. Spanning 4752 mile^2^, Los Angeles County is divided into 8 geographical Service Planning Areas (SPA) which enables the Department of Public Health to plan and implement targeted public health and clinical programs based on the needs of each area [17]. Thus, identification of areas of low medication access within the county is essential for the improvement of population health.

Although pharmacy deserts have been identified in various locales such as Chicago, New York City, Shelby County Tennessee, and the state of Pennsylvania [3, 5, 7, 15], to our knowledge, no study has identified and characterized pharmacy deserts in Los Angeles County. More importantly, existing studies have not fully characterized the types of areas that are more than 1 mile from the nearest retail pharmacy outlet. Previous research has identified deserts based on a compound definition adopted from food desert research that included certain predefined thresholds for poverty, vehicle access, and/or racial ethnic composition [3, 5]. By limiting the areas that are defined as deserts solely to predefined socioeconomic factors adopted from food desert literature, the full range of desert characteristics are not captured and may inappropriately exclude other areas that might be medication poor on the basis of other social factors, such as linguistic isolation, or access to health care professionals. Other research has utilized “1 mile away” from the nearest pharmacy as the only criteria, while not explicitly considering inherent difference between rural, urban, and suburban designations [4, 15]. It is important to address this gap in the literature by characterizing the nature of all areas that meet the 1-mile distance criteria, and by identifying the social factor typologies of pharmacy deserts. Thus, the main objective of this study was to identify and describe all pharmacy desert types found in Los Angeles County, using all areas 1 mile or greater away from a community pharmacy outlet, and then to explicitly characterize the types of deserts by differences in the social determinants of health indicators and by community pharmacy type.

## Methods

### Data Sources

#### Community Pharmacies

Pharmacy data was obtained from the California Department of Consumer Affairs Pharmacy Board Licensee database which was updated as of January 2020. Community pharmacies with an active license that were either retail chain franchises or independently owned were extracted from the database and included in the analysis.

#### Social Determinants of Health

Existing literature on food deserts and pharmacy deserts were used to determine the universe of SDOH variables for this study. Indicators such as poverty level, household ownership, vehicle ownership, education attainment, health insurance status, and language spoken at home were extracted from the 2012–2017 American Community Survey (ACS) for census tracts in LA County. United States census population data included total population, population aged 65 years and older, population aged 17 years and younger, and population by race and ethnicity. All ACS and population level data were reported as number of residents of the total population in the census tracts and the percent of total residents in the given category were calculated. Health Resources and Services Administration (HRSA) data regarding health professional shortage areas was also included as a binary SDOH indicator. Crime data was obtained from Relocation Essentials crime reports and was measured as a 1–10 integer with 5 being the national average index [19]. Thus, values less than 5 are lower than average, while those greater than 5 are higher than average. The HRSA and population level crime data was reported as an average of the indices within the census tracts.

#### Geo-statistical Analysis

Independently owned and retail chain pharmacies were geocoded and included in the analysis. Hospital and ambulatory clinic pharmacies were excluded as such pharmacies are often closed to the public. Street network driving distances were calculated from each census tract (2010) centroid in Los Angeles County to the nearest community pharmacy. Pharmacy deserts were defined as tract centroids that were more than 1 mile away through the road network to the nearest community pharmacy.

The census-based SDOH variables, HRSA health professional shortage area, and crime indices were merged with the tract deserts and K-means clustering was utilized to identify variation in pharmacy desert composition and typology and generate statistically relevant variable groupings. Geographical and statistical analyses were performed using ArcGIS 10.6 (ESRI, Redlands, CA).

#### Identifying SDOH Group Clusters and Mapping

A K-means algorithm was used for grouping the SDOH indicators of the pharmacy deserts. The goal of the K-means algorithm is to partition features so that the differences among the features in a group, over all groups, are minimized. Because the algorithm is non-deterministic polynomial-time (NP) “hard,” meaning all grouping combinations must be tested, a greedy heuristic is employed to group features. The greedy algorithm will always converge to a local minimum but will not always find the global (most optimal) minimum.

The K-means algorithm utilized in this study operates by first identifying random seed features used to grow each group. Consequently, the number of seeds will always match the number of groups. The procedure evaluates the fit of two groups, then 3 groups, then 4…, all the way up to 14 potential groups. The first seed is selected randomly. Selection of remaining seeds, however, while still employing a random component, applies a weighting that favors selection of subsequent seeds farthest in data space from the existing set of seed features (this part of the algorithm is called K-means ++). Once the seed features are identified, all features are assigned to the closest seed feature (closest in data space). For each cluster of features, a mean data center is computed, and each feature is reassigned to the closest center. The process of computing a mean data center for each group and then reassigning features to the closest center continues until group membership stabilizes (up to a maximum number of 100 iterations). The resulting clusters were then examined, graphed, and mapped.

## Results

### Pharmacy Deserts and Pharmacy Non-Deserts

**Fig. 1:**
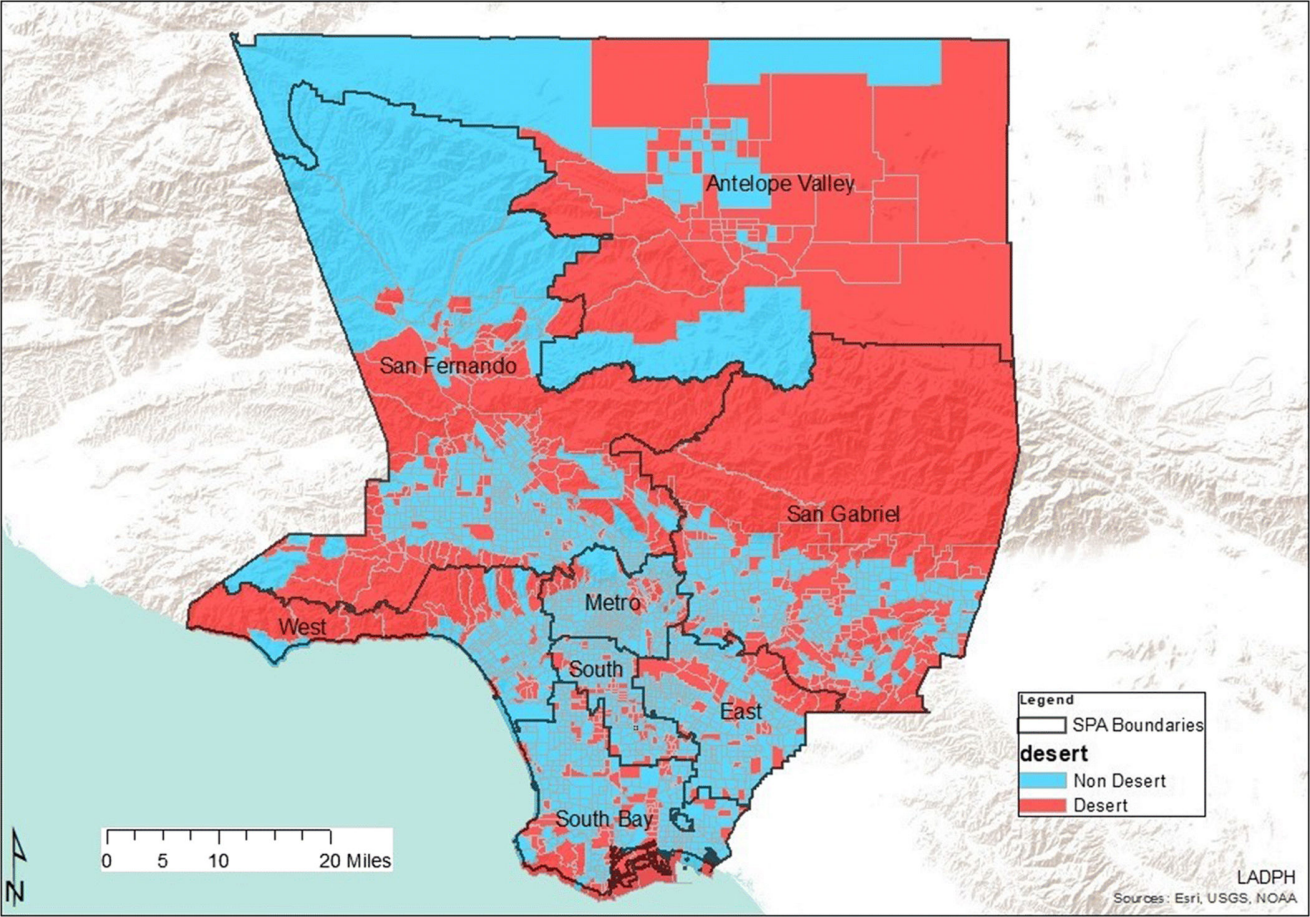
Los Angeles County pharmacy deserts and pharmacy non-deserts by Service Planning Area

The basic pharmacy desert criteria of the geographic accessibility of the census tract centroid (geometric center) being greater than 1 mile through the street network to a community pharmacy were used to identify and define 594 of 2346 census tracts as pharmacy deserts (Fig. 1). Twenty-three of these tracts had little or no population and were excluded from the analysis. Thus, as depicted in Table 1, 25% (571/2323) of LA County census tracts were pharmacy deserts and 75% (1752/2323) were pharmacy non-deserts. Furthermore, 24% (2,410,699/10,048,784) of LA County residents lived in tracts with centroids more than 1 mile from the nearest community pharmacy. The median street network distance to the nearest retail pharmacy for all residents living in pharmacy deserts was 1.38 miles, while the median distance for those that resided in pharmacy non-desert residents was 0.50 miles. Table 1 portrays the distribution of pharmacy deserts and pharmacy non-deserts in Los Angeles County by SPA. San Fernando (SPA 2) had the largest population and number of pharmacy deserts and pharmacy non-deserts overall. On the other hand, West (SPA 5) had the least number of pharmacy deserts and the second smallest population overall. Interestingly, while Antelope Valley (SPA 1) had the smallest population, it had more pharmacy deserts than SPA 5 whose population was almost two times more than the population of SPA 1. Antelope Valley also had the most deserts when compared to non-deserts in a SPA, overall. Finally, Metro (SPA 4) had almost 8 times less pharmacy non-deserts when compared to pharmacy deserts within the SPA even though its population was less than the population of San Fernando (SPA 2).

### Pharmacy Desert Social Determinants of Health Compositional Elements

The mean population density of all 571 pharmacy deserts was 4868 residents per square mile (Table 2). The number of residents that lived in a pharmacy desert that were non-Hispanic Black (NHB), non-Hispanic Asian (NHA), and non-Hispanic White (NHW) was 205,154, 341,973, and 708,095, respectively. Interestingly, there was an inverse relationship between NHW and NHA race and the pharmacy desert clusters. In other words, pharmacy deserts were characterized by having less NHW and NHA residents. Many of the deserts had a higher population of residents that identified as Hispanic, spoke English as a second language, and 306,202 residents did not have a high school diploma in all deserts combined. There were also more residents who were younger than 18 years of age that lived in a pharmacy desert when compared to residents that were 65 years of age or older. Overall, Los Angeles County pharmacy deserts had crime indices for property and people that were comparable to the nation average of 5. Residents who lived in deserts tended to not own their own vehicle or home and lived below the federal poverty line. Finally, pharmacy desert residents likely lacked health insurance and lived in areas of health professional shortages.

### Pharmacy Desert Sub-Types

**Fig. 2:**
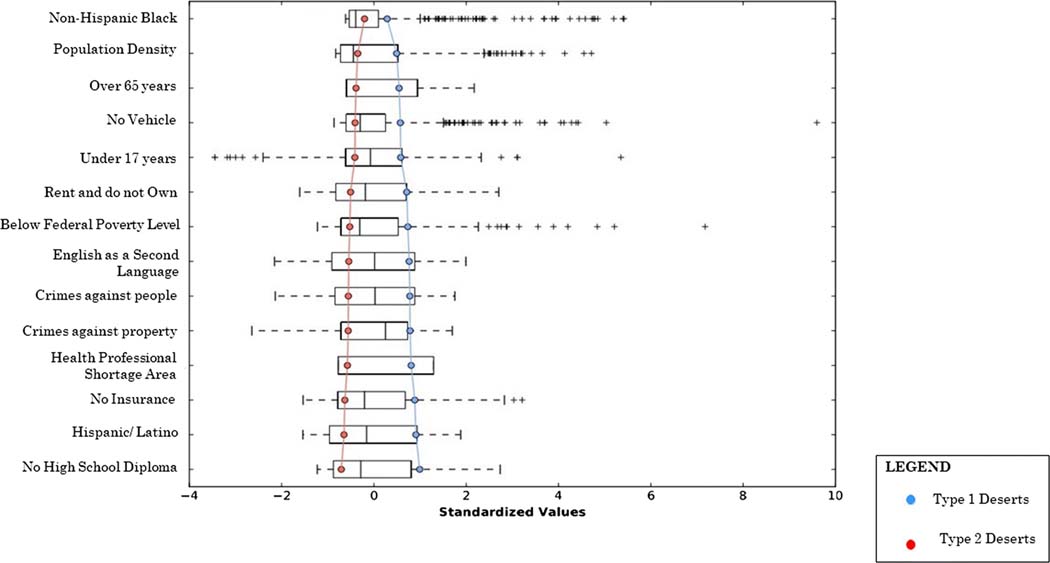
Parallel box plot of social determinants of health characteristics in pharmacy desert types

Two statistically independent groups of pharmacy deserts were yielded from the iterative K-means clustering algorithm (Table 3). Type one pharmacy deserts contained 238 census tracts and had a total population of 1,054,645 residents and type two pharmacy deserts contained 333 census tracts with a total population of 1,356,054 residents. Type one deserts differed from type two deserts regarding population factors associated with the SDOH principles of social and community context, economic stability, education, neighborhood environment, and health care (Fig. 2). Type one desert residents lived in much denser communities. The population density in type one pharmacy deserts was nearly 3 times more than the population density of type two deserts. Type one deserts were also characterized by twice as many African American and Hispanic residents. While the number of residents under the age of 18 was comparable in both type one and type two pharmacy deserts, there were two times more residents over the age of 64 that lived in type two pharmacy deserts.

The number of residents living below the federal poverty level in type one pharmacy deserts was nearly two times that of type two residents and a similar trend was seen regarding renting and not owning one’s own home. Furthermore, type one deserts had 3 times as many residents that did not have a high school diploma and almost one and a half times more residents that spoke English as a second language. Considering transportation, two times as many residents in type one deserts had no vehicle when compared to residents in type two deserts. While the average national index for crimes against property and people is 5, type one deserts encompassed areas with and indices that were almost one and a half times more than the national average. Type two pharmacy deserts encompassed areas with crime indices that were less than the national average. Additionally, type one pharmacy deserts had a health professional shortage index that was 8 times that of type two pharmacy deserts and there were twice as many type one desert residents that did not have health insurance than type two desert residents.

**Fig. 3:**
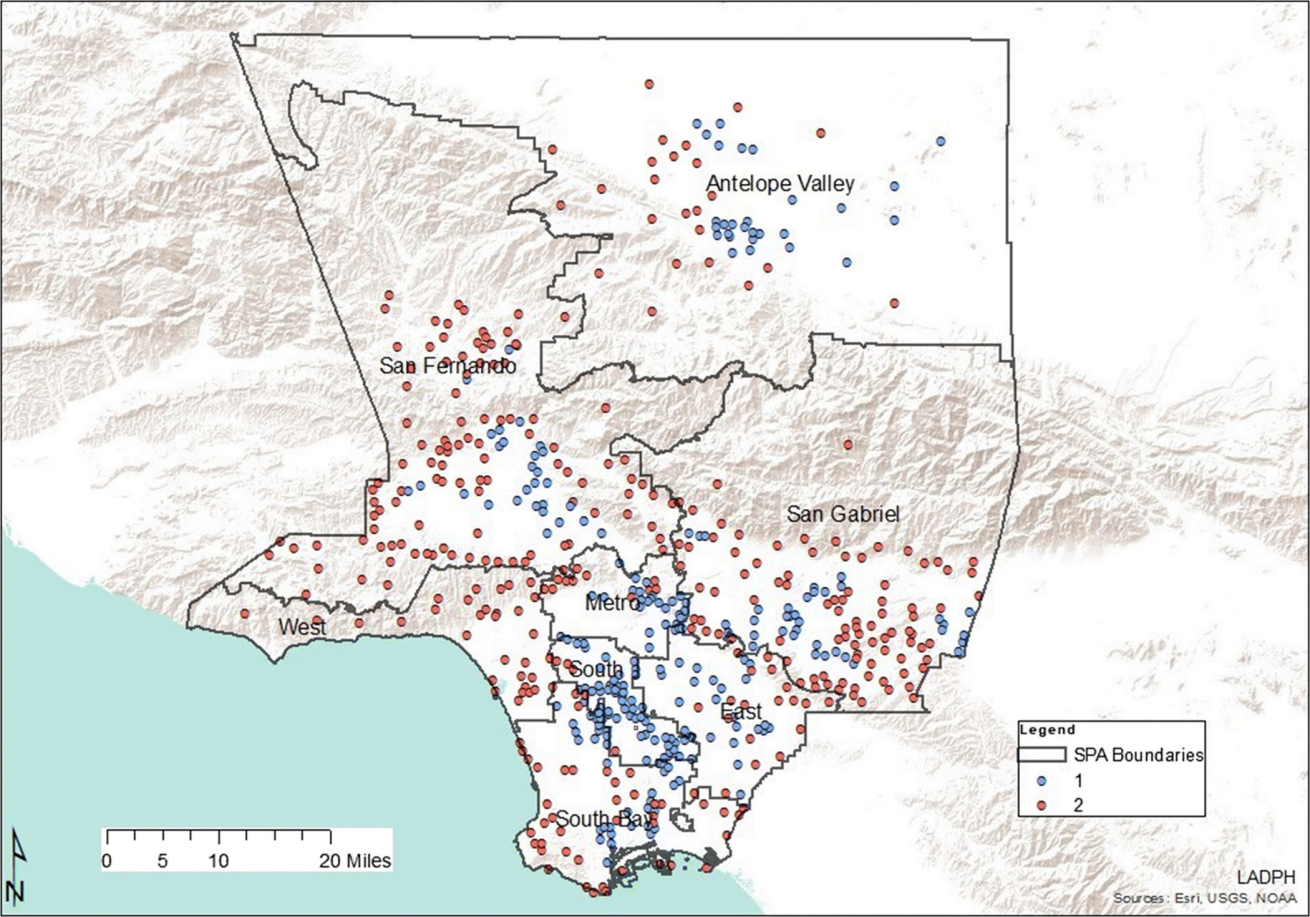
Pharmacy desert types by Service Planning Area

**Fig. 4:**
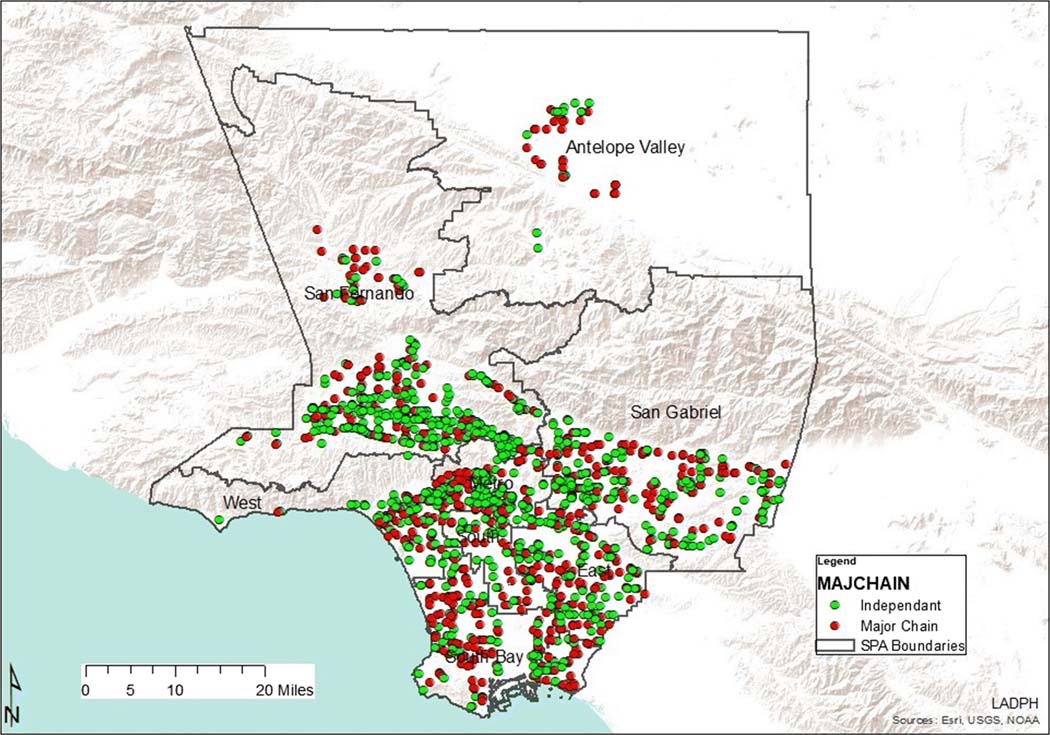
Los Angeles County community pharmacies by type and SPA

As demonstrated in Table 4, type one pharmacy deserts were most abundant in South LA communities (SPA 6), which also had the largest population density in Los Angeles County. There were two times fewer type one deserts in SPA 4, although its population density is the second highest in Los Angeles County (Fig. 3). On the contrary, the West had zero type one deserts as all pharmacy deserts in this area were type two. Type two deserts were most abundant in SPA 2 and least present in SPA 6. As depicted by Table 5 and Fig. 4, there were 1682 Los Angeles County community pharmacies extracted from the California Department of Consumer Affairs database and included in the analysis. Service Planning Area 4 had the most community pharmacies per 1000 residents while SPA 6 had the least community pharmacies per 1000 residents. Although SPA 6 had a comparable population density to SPA 4, the number of pharmacies per 1000 residents in SPA 4 was almost 7 times that of SPA 6.

## Discussion

Pharmacy deserts have been identified in various locales across the USA [3, 5, 7, 15]. To our knowledge, this study is the first to identify and characterize pharmacy deserts in Los Angeles County. Furthermore, this is the first study anywhere to specifically examine the internal composition of geographically defined pharmacy deserts using SDOH indicators and K-means clustering analysis. Our study revealed that LA County pharmacy deserts were of two distinct types (Fig. 3). Type one pharmacy deserts consisted of more residents that identified as non-Hispanic Black or Hispanic and less residents that identified as non-Hispanic White or non-Hispanic Asian. This corroborates earlier studies in which there was a disparity in pharmacy access in minority communities [3, 7]. Our study revealed that when compared to type 2 pharmacy deserts, more residents of type one deserts lived below the FPL, rented and did not own their own home, had no high school diploma, spoke English as a second language, had no vehicle, and lacked health insurance (Table 3). Moreover, type one deserts also encompassed areas with higher indices of crime and health professional shortage similar to previous works [3, 7].

These findings expand upon the definition adopted from the food desert literature and used in prior literature that have either described SDOH indicators in pharmacy deserts once the deserts were identified by geography or have used the basic greater-than-1-mile-travel-distancepharmacydesert definition along with the pre-determined SDOH indicators from the nutritional desert literature to characterize pharmacy deserts [3, 5, 7]. However, both research approaches have inherent problems that limit the interpretability of their results. The first approach does not facilitate the true discernment of contextual-based differences in all the places that are more than 1 mile from community pharmacies, thus leading to potential conflation between rural, suburban, and urban environments. The second approach based on food desert indicators is self-limiting and misses relevant indicators (i.e., health care provider shortages) that do not meet the specified non-distance-based exclusion criteria. The results of this study differ and extend the literature as the basic geographic definition of pharmacy deserts was used and merged with SDOH indicators followed by application of K-means clustering analysis to yield the 2 distinct pharmacy desert cluster sub-types.

The causes of divergent SDOH factors in types 1 and 2 pharmacy deserts (Fig. 2) have a historical formation in structural inequity which contributes to population migration in Los Angeles County. Prior to the Civil Rights Movement, exclusionary practices such as racial zoning and redlining maintained NHB and Hispanics in impoverished, densely populated neighborhoods, while NHW out-migrated to suburban areas [20, 21]. While redlining no longer exists, race riots, economic forces which have increased housing costs, and immigration all contribute to the persistence of predominantly minority communities in Los Angeles County [22]. More specifically, predominantly minority communities are in SPA 6, which consists of 68% Latinos and 27% African Americans and in SPA 7, which is 74% Latino [17]. Furthermore, predominantly NHW communities of white out-migration are in SPA 5 (64% White) and SPA 2 (45% White) [17]. Structural inequity factors regarding minority race/ethnicity and low socioeconomic status are also associated with low education attainment, high crime rates, and poor access to health care [23–27]. Altogether, our findings suggest that living in a type one pharmacy desert likely compounds limited community pharmacy access due to competing needs. While residents of type two pharmacy deserts might lack access to community pharmacies solely based on the travel distance, more of these residents also have vehicles, and live in suburban areas where the community pharmacies might be spread farther apart geospatially by design since the population density is almost 3 times less than that of type one pharmacy deserts.

As Los Angeles County is diverse, pharmacy deserts and non-deserts varied in each Service Planning Area across the county (Fig. 1). This aligns with a previous study that demonstrated differential geographic access to community pharmacies in New York communities of varied socioeconomic levels [5]. It is not surprising that SPA 2 would have the most pharmacy deserts, community pharmacies, and independently owned pharmacies, based on population size (Table 1 and Table 5). San Fernando also had the most type two pharmacy deserts. This suggests that market factors such as consumer demand, health insurance coverage, health professionals, and competitor pharmacies in area might drive pharmacy access in this SPA. Health care access for residents that live in in these types of deserts might be also be considered less burdensome. In other words, there is an increased chance of profit where there are more potential customers, especially customers that are likely to have prescription drug coverage (health insurance). More residents of type two pharmacy deserts have health insurance when compared to those living in type one deserts. Regarding situational analysis, owners of independent pharmacies often open new pharmacies close to medical offices with the intention of building relationships with health care providers in nearby medical practices [28]. Type two pharmacy deserts encompassed less HPSA than type one pharmacy deserts. Furthermore, opening a pharmacy at an optimal location, one that is intentionally located farther away from competitors, likely benefits profit margins. This is supported by past studies in which pharmacy owners were found to make decisions about market entry and exit based on population density and community income status [15, 29, 30]. It is crucial to note here that while SPA 5 pharmacy deserts were all type two deserts, there were only four type two deserts in SPA 6, the innercity area.

Ninety-three percent of all pharmacy deserts in SPA 6 were type one deserts. Service Planning Area 6 also had the second smallest number of independent and chain pharmacies following SPA 1 and the least pharmacies per 1000 residents, when compared to the remaining 7 SPAs. However, there were 51% more residents per square mile living in SPA 6 than SPA 1. This is significant as it suggests that residents in SPA 6 lack access to much needed pharmacy services such as immunizations, pre-exposure, and post-exposure prophylaxis for human immunodeficiency virus (HIV), tobacco cessation assistance, contraception assistance, medication management, and naloxone [31]. For example, a recent systematic review of pharmacists’ effect on older adults’ access to vaccines in the USA revealed that pharmacists positively impacted older adults’ access to pneumococcal and influenza vaccinations [32]. Studies have also reported the lack of community pharmacy services such as 24-hour access, drive through, on site clinics, and delivery in low-income minority communities [2, 15, 33]. Finally, as type one pharmacy deserts comprise areas with higher indices of crime against people and property, it is likely that this may cause pharmacy stakeholders not to build independently owned or retail chain community pharmacies in the area.

Our findings are especially important because low-income minority communities often have the highest prevalence and incidence of chronic conditions. According to the Los Angeles County Department of Public Health 2017 Key Health Indicators, SPA 1 and SPA 6 had the highest percentages (14% and 12%, respectively) of adults ever diagnosed with diabetes and fared worse along with SPA 6, SPA 7, and SPA 4 regarding the age-adjusted diabetes death rate (32, 38, 26, and 24 deaths per 100,000 population, respectively). Antelope Valley also had the highest percentage of adults diagnosed with hypertension (30%) and ranked first, followed by SPA 6 and SPA 8 regarding the age-adjusted coronary heart disease death rate (149, 148, and 122 deaths per 100,000 population, respectively) [17]. A similar pattern emerged with the age-adjusted stroke death rate, and COPD/emphysema and pneumonia/influenza mortality rates as SPA 6, SPA 1, and SPA 8 fared worse for of these diseases when compared to the remaining 5 SPAs. In addition, SPA 6 had the lowest vaccination percentages for influenza and pneumonia and SPA 1 had the highest percentages of smokers [17]. Medications play a vital role in primary, secondary, and tertiary prevention of each of the aforementioned public health issues. Given the preventive services that pharmacies provide, coupled with the fact that pharmacies are often the most accessible source for health care within the community, be it through delivery or 24-hour service, pharmacy deserts pose a threat to, and worsen community health and wellness outcomes [34].

This study has several limitations. First, we did not assess community pharmacy characteristics that impact access such as home delivery, medication shipping/mail order or 24-hour access. We also used census tract centroids as a proxy for residential address data to calculate travel distances. Furthermore, an assumption was made regarding medication procurement behavior: that the nearest community pharmacy to residents was their actual pharmacy home. Some residents might travel to a pharmacy outside of their community to procure their medications or use clinic or outpatient hospital pharmacies. Moreover, residents who live at or near the LA County boundaries might also travel to pharmacies in neighboring counties. While health indicators regarding disease burden in LA County were included, we did not include information on corresponding prescription medication availability in pharmacies. Finally, we only examined community pharmacies in LA County; thus, our results are not generalizable to other locations nationally or internationally. Nevertheless, this study extends the literature regarding pharmacy deserts in the USA. We excluded clinic-based and outpatient hospital pharmacies which often serve a closed target population. This allowed us to include the entire county population and served as a surrogate for their pharmacy use and medication procurement behavior at the community level. Furthermore, the utilization of an iterative K-means clustering analysis to characterize pharmacy deserts allowed for deserts that were more representative of diverse population of Los Angeles County.

Future research should investigate the associations between medication adherence and access and pharmacy deserts in LA County, specifically in poor minority communities. Researchers should also examine patient perspectives of medication procurement and medication use behavior in under-resourced settings. New research should explore the availability of medications in pharmacies as they pertain to disease burden and health outcomes within the county as this can inform policy. More specifically, such research can lead to federal funding to develop, implement, and evaluate equity-based programs that, in LA County, are tailored for each Service Planning Area, and increase access to pharmacies and medication for pharmacy desert residents. As limited community pharmacy access also has a business perspective, pharmacy business and local and federal stakeholders should also consider the social determinants of health when planning market entry. Clinicians should consider federal funding for implementation of interdisciplinary, team-based, innovative programs and networks that increase pharmacy and medication access for their patients. Finally, clinicians that serve poor minority communities should leverage community engagement and collective action to increase residents’ access to pharmacies and medications.

## Figures and Tables

**Table 1:** Pharmacy deserts and non-deserts in LA County Service Planning Areas

Service Planning Area (SPA)/number	Pharmacy deserts (*N*)	Pharmacy^*^ non-deserts (*N*)	Total census tracts	Total population
Antelope Valley (1)	52	32	84	392,683
San Fernando (2)	136	378	514	2,212,327
San Gabriel Valley (3)	126	265	391	1,787,632
Metro (4)	36	284	320	1,153,736
West (5)	32	129	161	656,483
South (6)	58	170	228	1,032,551
East (7)	46	242	288	1,310,864
South Bay (8)	85	275	360	1,502,508
Total	571	1752	2346^*^	10,048,784

* 23 census tracts with no population excluded | total number of census tracts 2323

**Table 2:** Social determinants of health elements in all pharmacy deserts

		Population characteristic	Residents (*n*)	Share
Healthy People 2020 SDOH Framework	Social and community context	Population density	4868^*^	0.17
		Hispanic	1,100,240	0.59
		Non-Hispanic Black	205,154	0.06
		Younger than 18 years of age	574,445	0.24
		Older than 64 years of age	327,851	0.21
	Economic Stability	Below federal poverty level	341,058	0.38
		Rent and do not own home	891,140	0.36
	Education	No high school diploma	306,202	0.70
		English as a second language	1,153,051	0.45
	Neighborhood and environment	No vehicle	42,027	0.23
		Crimes against property	5.48^**^	0.44
		Crimes against people	4.95^**^	0.43
	Health care	Health professional shortage area	0.37^**^	0.46
		No health insurance	574,445	0.56

* Residents per square mile

** Mean index | characteristics are for all 571 pharmacy deserts in total

**Table 3:** Social determinants of health elements by pharmacy desert type

			Type 1 desert (*N* = 238)		Type 2 desert (*N* = 333)	
		Population characteristic	Residents (*n*)	*R* ^2^	Residents (*n*)	Share
Healthy People 2020 SDOH Framework	Social and community context	Population density	7753^*^	0.99	2806	0.65
		Hispanic	737,380	0.82	362,410	0.94
		Non-Hispanic Black	126,402	0.92	78,749	1.00
		Younger than 18 years of age	289,409	0.54	285,036	1.00
		Older than 64 years of age	100,522	1.00	227,329	1.00
	Economic stability	Below federal poverty level	237,266	0.71	103,792	1.00
		Rent and do not own home	549,198	0.99	341,942	0.96
	Education	No high school diploma	224,904	0.86	81,298	0.63
		English as a second language	652,985	0.83	500,066	0.85
	Neighborhood and environment	No vehicle	26,884	1.00	15,143	0.38
		Crimes against property	7.10^**^	0.67	4.32	0.89
		Crimes against people	6.74^**^	0.78	3.67	0.89
	Health care	Health professional shortage area	0.76^**^	1.00	0.10	1.00
		No health insurance	143,906	1.00	71,230	0.74

* Residents per square mile

** Mean index

**Table 4:** Pharmacy deserts in LA County Service Planning Areas by desert type

SPA number	Type 1 deserts (*N*)	Population (residents)	Type 2 deserts (*N*)	Population (residents)	Population density^*^	Geographic description
Antelope Valley (1)	28	149,831	24	101,113	282	Rural
San Fernando (2)	28	135,349	108	441,605	2104	Suburban
San Gabriel Valley (3)	36	165,382	90	378,883	2205	Suburban
Metro (4)	25	90,818	11	39,068	12,588	Urban
West (5)	0	0	32	121,193	3349	Mixed^**^
South (6)	54	239,858	4	15,821	14,430	Urban
East (7)	26	116,685	20	100,023	8244	Suburban
South Bay (8)	41	156,722	44	158,348	4586	Mixed^**^
Total	238	1,054,645	333	1,356,054		

* Residents per square mile

** Mixed= urban and suburban

**Table 5:** Community pharmacies in LA County Service Planning Areas by pharmacy type

SPA number	Independent pharmacies (*N*)	Chain pharmacies (*N*)	Total pharmacies (*N*)	Population density^*^	Pharmacies per 1000 residents
Antelope Valley (1)	14	30	44	282	0.17
San Fernando (2)	306	159	465	2104	0.80
San Gabriel Valley (3)	186	134	320	2205	0.59
Metro (4)	147	79	226	12,588	1.74
West (5)	93	56	149	3349	1.22
South (6)	36	31	67	14,430	0.26
East (7)	97	93	190	8244	0.88
South Bay (8)	94	127	221	4586	0.70
Total	973	709	1682		

* Residents per square mile
